# Investigating Misophonia: A Review of the Empirical Literature, Clinical Implications, and a Research Agenda

**DOI:** 10.3389/fnins.2018.00036

**Published:** 2018-02-07

**Authors:** Jennifer J. Brout, Miren Edelstein, Mercede Erfanian, Michael Mannino, Lucy J. Miller, Romke Rouw, Sukhbinder Kumar, M. Zachary Rosenthal

**Affiliations:** ^1^International Misophonia Research Network, New York, NY, United States; ^2^Department of Psychology, Center for Brain and Cognition, University of California, San Diego, San Diego, CA, United States; ^3^Department of Psychology and Neuroscience, Maastricht University, Maastricht, Netherlands; ^4^Center for Complex Systems and Brain Sciences, Florida Atlantic University, Boca Raton, FL, United States; ^5^Department of Psychology, Brain and Cognition, Amsterdam University, Amsterdam, Netherlands; ^6^Brain and Cognition, Department of Psychology, University of Amsterdam, Amsterdam, Netherlands; ^7^Auditory Group, Institute of Neuroscience, Newcastle University, Newcastle, United Kingdom; ^8^Department of Psychiatry and Behavioral Science, Duke University Medical Center, Durham, NC, United States; ^9^Department of Psychology and Neuroscience, Duke University, Durham, NC, United States

**Keywords:** misophonia, sensory processing, sensory over-responsivity, fear circuitry, defensive motivational systems, emotion regulation

## Abstract

Misophonia is a neurobehavioral syndrome phenotypically characterized by heightened autonomic nervous system arousal and negative emotional reactivity (e. g., irritation, anger, anxiety) in response to a decreased tolerance for specific sounds. The aims of this review are to (a) characterize the current state of the field of research on misophonia, (b) highlight what can be inferred from the small research literature to inform treatment of individuals with misophonia, and (c) outline an agenda for research on this topic. We extend previous reviews on this topic by critically reviewing the research investigating mechanisms of misophonia and differences between misophonia and other conditions. In addition, we integrate this small but growing literature with basic and applied research from other literatures in a cross-disciplinary manner.

## Introduction

Misophonia is a complex neurophysiological and behavioral syndrome characterized by heightened physiological responsivity and a high magnitude of emotional reactivity resulting from intolerance to specific auditory stimuli (Jastreboff and Jastreboff, 2001, 2014; Møller, 2011; Wu et al., 2014). Originally described by Jastreboff and Jastreboff (2001), individuals with misophonia are believed to demonstrate increased sympathetic nervous system arousal, accompanied by emotional distress in response to specific pattern-based sounds, irrespective of decibel level (Jastreboff and Jastreboff, 2001; Edelstein et al., 2013). Examples of these sounds include other people chewing, throat clearing, slurping, finger tapping, foot shuffling, keyboard tapping, and pen clicking (Jastreboff and Jastreboff, 2001; Edelstein et al., 2013; Schröder et al., 2013; Wu et al., 2014). The acoustic pattern of these sounds and their elicited response vary across individuals. Both sounds and reactions appear to take on idiosyncratic forms, suggesting that individual differences, learning and context may play a role in aversive responding.

Sounds are referred to as “triggers” and as “misophonic sounds” by sufferers in social media support forums and by researchers in the emerging scientific literature. Similarly, responses to trigger sounds are often referred to as “misophonic responses.” Upon exposure to misophonic trigger sounds, emotional responses frequently include anger (ranging from irritation to rage), anxiety, disgust, avoidance, escape behavior as well as a feeling of being overwhelmed and/or overloaded by auditory stimuli. As noted in the nascent literature, this newly defined syndrome may, for some individuals, lead to severe impairments in daily functioning (e.g., occupationally, interpersonally, academically) and may contribute to the development of behavioral health problems.

Although syndromal features have begun to be characterized empirically, misophonia has not been formally recognized as a specific type of neurological, audiological, or psychiatric disorder. Over-responsivity to auditory stimuli is a feature observed among a wide range of neurological, auditory, medical and psychiatric disorders such as tinnitus, hyperacusis (Jastreboff and Jastreboff, 2001), migraine headaches (Sullivan et al., 2013), autism spectrum disorder (Ben-Sasson et al., 2009a; Danesh and Kaf, 2012; Lane et al., 2012), posttraumatic stress disorder (Attias et al., 1996; Finsterwald and Alberini, 2014), borderline personality disorder (Rosenthal et al., 2016), bipolar disorder, and schizophrenia (Cabranes et al., 2013). The precise nature of the relationship between misophonia and these disorders is unknown. However, intolerance to aversive sounds does not appear to be a phenomenon that co-occurs uniquely and specifically with any one disorder. Indeed, rigorously conducted research is needed to elucidate whether misophonia is a unique constellation of symptoms or a transdiagnostically co-occurring syndrome found across other disorders (Stansfeld et al., 1985).

The small body of research investigating misophonia includes studies conducted in the fields of audiology, otolaryngology, psychiatry, psychology, and the neurosciences. Although some promising research examining the neurobiological underpinnings of misophonia recently has been conducted, much of the early literature describes the phenotypic expression and preliminary associations between symptoms of misophonia and psychiatric disorders.

The primary aim of this paper is to review research on misophonia using a cross-disciplinary approach, with the goal of generating testable hypotheses and advancing the conceptualization of this recently identified syndrome. In addition, recent neuroscience-based paradigms of emotion emphasizing the defense/fear circuitry are described to contextualize the extant research and inform future studies. Specifically, we begin by detailing Jastreboff and Jastreboff (2001) original theoretical model of misophonia and related conditions. Next, we review the current research literature on misophonia, with a brief discussion of the early case studies and the small number of empirical studies that have followed. Last, we synthesize the empirical literature, outline a research agenda, and highlight several key considerations in the treatment of those with misophonia.

## Origin of misophonia: the Jastreboffs' model

In this section, we trace the development of Jastreboff and Jastreboff's (Jastreboff and Jastreboff, 2001) conceptualization of misophonia. Although other models of sound intolerance have been posited (Pienkowski et al., 2014; Tyler et al., 2014), misophonia as a specific syndrome was initially described by the Jastreboff's (Jastreboff and Jastreboff, 2001). The model began with Pawel Jastreboff's phantom model of tinnitus (Jastreboff, 1990) and was influenced by Jastreboff's work with hyperacusis (Jastreboff and Jastreboff, 2001, 2002, 2014). Tinnitus is a complex neurological condition with multiple potential etiological pathways (Baguley, 2016). Individuals with tinnitus experience ringing in one or both of their ears, and the condition often is associated with hearing loss. Jastreboff's neurophysiological model of tinnitus as a phantom perception gained wide acceptance when he published it in 1990 (Baguley, 2016). According to this theory, tinnitus arises due to abnormal patterns of neural activity in auditory neural pathways. These abnormal activity patterns, possibly originating from the cochlea (or in the subcortical structures in the auditory neurological pathway) are detected by the neocortex where they are perceived as a ringing noise. Essential to this model of tinnitus is the difference between perception of tinnitus *per se* and emotional responses to these symptoms. Reacting negatively to tinnitus facilitates attention to it, which further amplifies the percept of tinnitus. With repetition of such responses, associative learning between tinnitus and these negative emotional responses occurs. In other words, whereas the perception of tinnitus involves the auditory system, the emotional response to tinnitus is modulated by a wider range of neural systems, including those within the limbic areas. Based on this, Pawel and Margaret Jastreboff developed Tinnitus Retraining Therapy (TRT), which includes repeated exposure to low level, broadband noise in an effort to facilitate habituation by interfering with the neural activity responsible for generating tinnitus (Jastreboff et al., 1996).

Using a similar model, Jastreboff and Jastreboff (2001) purported that anatomical and functional links between the central auditory system and the limbic system were integral to the development of fear and anxiety related to hyperacusis (i.e., extreme sensitivity to loud noise or the perception that noises are much louder than they are). This model has been supported by observations that patients with hyperacusis may present without any apparent dysfunction or involvement within the peripheral auditory apparatus (Hazell and Jastreboff, 1990). In addition, Jastreboff suggested that because of hyperacusis, many individuals also develop phonophobia (i.e., a pronounced fear of sound). As with tinnitus, the Jastreboffs' (Jastreboff and Jastreboff, 2001, 2002, 2014) proposed using the same principles of TRT to help individuals with hyperacusis and phonophobia (Baguley and McFerran, 2011).

Jastreboff and Jastreboff (2001) coined the term misophonia while working with hyperacusis patients. They noted that some hyperacusis patients reacted with aversion to sounds that have specific patterns regardless of decibel level and irrespective of the physical characteristics of sounds (Jastreboff and Jastreboff, 2001). The Jastreboffs reported (Jastreboff and Jastreboff, 2001) that the sounds to which misophonics responded included, as examples, slurping, lip smacking, breathing, and pencil tapping. In addition, unlike hyperacusis, misophonic triggers were variable across people and environmental contexts. Consequently, the Jastreboffs (Jastreboff and Jastreboff, 2001) hypothesized that these responses were developed and maintained, in part, via associative learning mechanisms activated in particular contexts. Accordingly, it could be hypothesized from this framework that misophonic responses to trigger cues were grounded in neurophysiological systems responsible for emotion, memory, and learning. From this perspective, misophonic responses to sounds may be inherently both biological and shaped by environmental influences.

Thus, with higher cortical brain structures involved in the maintenance of misophonia, the Jastreboff's used the framework of TRT to propose a treatment approach for misophonia. Specifically, they proposed a treatment model using repeated exposure to misophonic triggers with new and positively experienced conditioned responses temporarily following exposure to triggers. To date, this retraining intervention has not been experimentally tested using randomized controlled trials, leaving the efficacy of this treatment without empirical evidence.

## Case studies of misophonia

Most of the published literature exploring misophonia has been conducted using individual case descriptions or a series of case reports among small samples of adults self-reporting symptoms (Neal and Cavanna, 2012; Bernstein et al., 2013; Ferreira et al., 2013; Johnson et al., 2013; Kluckow et al., 2014; Webber et al., 2014; Dozier, 2015). Across these case studies, the specific symptoms vary. Although the Jastreboffs' original conceptualization of misophonia included aversive responding to sounds generated by both living beings and inanimate objects, many case reports specifically indicate that trigger sounds are generated by other people (e.g., other people chewing, smacking lips, coughing, throat clearing; Webber et al., 2014). However, it is important to note that individuals also report aversion to mechanical noises, such as air-conditioners, refrigerator humming, and/or noises emanating from pets (Møller, 2011; Cavanna and Seri, 2015). In addition, some case studies indicate that individuals with misophonia describe experiencing aversive responses to repetitively presented visual stimuli or movement, also known as misokinesia (e.g., seeing another person shaking their leg).

These case reports have been valuable as initial documentation of misophonia as a clinical syndrome not previously described and found among patients presenting with other clinical problems. For example, Neal and Cavanna (2013) observed misophonia symptoms in one patient with Tourette's syndrome. Webber et al. (2014) reported misophonia symptoms in a pediatric patient with Tourette's syndrome and obsessive compulsive disorder (OCD). As another example, using a case series approach with three patients, Ferreira et al. (2013) reported misophonia symptoms in patients with several different psychiatric disorders. Based on these case observations, Ferreira et al. (2013) speculated that misophonia could be characterized as a symptom of obsessive-compulsive disorder, generalized anxiety disorder (GAD), and schizotypal personality disorder. It is useful to generate hypotheses from case descriptions. However, it is premature to use such methods to draw causal inferences or to extrapolate results beyond the cases being described. For more definitive insights to be drawn from the empirical literature, appropriately powered studies are needed using experimental methods with testable hypotheses to elucidate the mechanisms underlying misophonia.

## Physiological measures and misophonia

### Autonomic, neurophysiological, and neurobiological studies

A small number of studies have investigated the relationship between subjective and behavioral responses in misophonia and corresponding responses in the brain and nervous system. These studies are the first to begin examining whether misophonic individuals respond in specific ways to misophonic trigger sounds as compared to other aversive sounds. Importantly, these studies have begun to identify candidate neural and peripheral psychophysiological mechanisms underlying misophonia.

Edelstein et al. (2013) were the first to apply psychophysiological measurements to study misophonia. They measured skin conductance response (SCR) to quantify sympathetic nervous system reactivity in misophonic and control participants using both unisensory^1^ and multisensory^2^ stimuli. The researchers used stimuli that ranged in emotional valence (e.g., children laughing, birds singing, gum chewing and lip smacking) and asked participants to rate each by their perceived level of aversion. These self-reports were compared with the physiological data.

First, the subjectively reported autonomic (i.e., “fight/flight”) response was present in the SCR data. Furthermore, the response was specific. Both aversiveness ratings and SCR data showed increased responses in auditory-only stimuli in misophonics as compared with controls, while no significant difference was obtained in visual-only stimuli. Results showed a significant positive correlation between average level of aversiveness and mean SCR across all participants and across unisensory and multisensory trials. This finding suggests that subjective and physiological responses to stimuli were consistent with one another. Overall, the importance of this study is that it indicates (a) misophonic responses can be measured in the autonomic nervous system and (b) misophonia is associated with heightened SCR responses to misophonic cues.

The authors note that limitations of the study include small sample size, a lack of rigorous screening for psychiatric or psychological problems, and the fact that SCR measures autonomic arousal but does not describe the nature of the affective state associated with autonomic arousal. In addition, because this study lacked a clinical comparison group, it is unclear whether the findings can be attributed to misophonia specifically, or to other clinical conditions. Indeed, recommended improvements for future studies using these methods includes (a) a larger sample size, (b) use of a clinical control group, (c) use of psychometrically validated and structured psychiatric diagnostic evaluations, (d) more specific probing of trigger sounds (e.g., including a trigger vs. non-trigger contrast in the analyses), and (e) use of dependent measures that can more clearly specify and differentiate exact physiological or subjective responses elicited by trigger sounds.

Edelstein et al. (2013) suggest that the potential underlying mechanisms of misophonia may bear some similarities with those of synesthesia and suggest that the two conditions may inform one another. Synesthesia is a condition in which a sensory stimulus or sensation (also known as an “inducer”) consistently and automatically evokes another seemingly unrelated sensation or association (also known as a “concurrent”; Ward and Simner, 2003; Saenz and Koch, 2008; Brang et al., 2010, 2011; Colizoli et al., 2013). Though unusual, it is possible for emotion to be a synesthetic concurrent, as indicated in previous research on tactile-emotion synesthesia (Ramachandran and Brang, 2008). A difference between synesthesia and misophonia, however, is that synesthetes have a more specific and complex set of inducer-to-concurrent associations (e.g., in a letter-color synesthete, the letter “A” may be blue while the letter “R” is purple). Furthermore, synesthetic responses remain constant over the years, and it is not yet clear if this is the case for misophonic responses. Still, the knowledge acquired in the past decade on the mechanisms involved in synesthesia does offer a useful preliminary model for misophonic mechanisms. Specifically, Edelstein et al. suggest that akin to the abnormal brain connectivity between inducer and concurrent brain areas obtained in synesthetes (Rouw and Scholte, 2007), deviant anatomical or functional connections could lie between auditory and limbic regions in misophonics.

Schröder et al. (2014) published the first EEG study on the neurobiological mechanisms involved in misophonia. The authors examined auditory event-related potentials (ERPs), including the P1, P2, and N1 components to explore the early auditory processing system in participants with misophonia. Notably, the N1 component is often associated with auditory attention and abrupt changes in the detection of sounds. Schröder et al. presented an oddball paradigm, wherein the participant listened to a sequence of standard tones, with deviant tones randomly interspersed. Based on research indicating that attention processing anomalies correlate with several psychiatric disorders, Schröder et al. hypothesized that similar atypical responses would be observed in misophonics. Indeed, the N1 ERP peak evoked by the oddball tones was diminished in misophonics, as compared to that of controls. The misophonics did not differ from controls in the P1 and P2 components of misophonics and controls during oddball tones, nor in any of the ERP components during standard tones.

Schröder and colleagues suggest that the observed N1 response may be a candidate neurophysiological marker for pathology related to misophonia. Although these results do not establish a definitive causal link between diminished N1 and misophonia, they do represent an important early step toward understanding the neural underpinnings of misophonia. Specifically, this study suggests how misophonia may affect early auditory processing components. The findings furthermore suggest a role for atypical auditory attentional processes in misophonia. As a limitation of the study, the authors note that the diminished N1 does not reveal the nature or level of the atypical processes. Further, the study results do not disconfirm the hypothesis that diminished N1 responding may reflect transdiagnostic impairment, rather than being unique to misophonia *per se*. Accordingly, it is premature to conclude that atypical N1 responses are specific to misophonia. A study contrasting electrophysiological responses in misophonics with other clinical control groups is needed to address this issue.

A technique commonly used to offer insight in the exact location of brain processes (due to higher spatial resolution) is functional magnetic resonance imaging (fMRI). A recent study by Kumar et al. (2017) performed a functional neuroimaging as well as psychophysiological [heart rate (HR) and galvanic skin response (GSR)] study with misophonic and age-matched controls. Participants were presented with three sets of sounds: trigger sounds, unpleasant sounds (aversive non-misophonic sounds) and neutral sounds. As expected, trigger sounds evoked a strong misophonic reaction in misophonic participants. The unpleasant sounds, although perceived as annoying, did not trigger a misophonic reaction, indicating a dissociation between general annoyance and misophonic responses.

The group (misophonic vs. control) by sound type (misophonic, unpleasant, neutral) interaction was significant in the bilateral anterior insular cortex (AIC). Specifically, the misophonics showed increased activation in this region in response to trigger sounds. No differences between misophonics and controls were found for the unpleasant and neutral sounds. Activity in bilateral AIC furthermore correlated with mean misophonic ratings, with increased scores related to increased activation. Accordingly, one important conclusion from this study is that the AIC is a neural structure that may have a key role in the processing of misophonic triggers.

The AIC is known to be a core hub of the “salience network” (Seeley et al., 2007) which detects personally relevant stimuli in the environment and directs attention to these cues. In the Kumar et al. (2017) study, stronger activation of AIC to trigger sounds show that misophonic participants assigned higher salience to trigger sounds. Analysis of functional connectivity of AIC showed hyper-connectivity, which was again specific to trigger sounds, to the default mode network (DMN) (Raichle et al., 2001) in misophonic participants. The DMN is known to be active during internally directed thoughts and recall of memories. Stronger coupling of AIC to DMN in misophonic participants suggests that processes related to associative learning and memory may have an important role in the heightened AIC activation to trigger sounds.

In addition, analysis of structural brain data in Kumar et al. (2017) showed that misophonics had greater myelination in the gray matter of ventromedial prefrontal cortex (vmPFC), which forms a node of the DMN. This structural difference possibly underlies the abnormal functional connectivity of AIC to DMN in misophonics. Finally, Kumar et al. observed heightened autonomic responses (HR and GSR) specific to trigger sounds in misophonic participants, and the analysis of sources of these responses were localized in AIC areas.

Collectively, the findings from Kumar et al. point to the abnormal activation and functional connectivity of AIC, shedding light on candidate regions and systems representing the possible neural underpinnings of misophonia. Ultimately, these findings may have clinical significance by offering clinical scientists key insights about possible biological mechanisms which can be targeted for change when developing ways to help people with misophonia.

An important limitation of this study is the lack of a clinical control group. Without comparing misophonic responses to those of individuals without misophonia but with other clinical characteristics, it is not possible to conclude that the findings from this study are unique and specific to misophonia. Another limitation of this study is that obtaining correlations and relationships among neural patterns of activation and behavior does not warrant causal interpretations. Additionally, in neuroimaging the problem of “reverse inference” makes it difficult to assign a specific and unique function to observed brain activity. Literature showing how a certain cognitive process leads to activation in a particular neural area, for example, does not validate the conclusion that activation in that brain area always reflects that particular cognitive function. However, Kumar et al. (2017) partially counters the latter issue by combining different techniques (neuroimaging, physiology, behavior) so that the different analyses can create converging evidence on the same interpretation and outcome.

Several conclusions can be drawn when considering, collectively, the small number of reviewed studies using autonomic, neurophysiological, and neurobiological measures. First, the subjective responses from misophonics are corroborated by physiological measurements of increased autonomic arousal in response to misophonic triggers. This research validates the experience of sufferers of misophonia by demonstrating that, indeed, misophonic cues do elicit automatic sympathetic arousal and negative affective states. Similarly, misophonics show atypical neuronal and physical responses in the brain and nervous system in response to their triggers. Second, current studies indicate a certain degree of specificity: responses to misophonic stimuli are different from “normal” aversive stimuli. The studies also point at a special role for central auditory processing impairments. Although this is an interesting notion, the effects observed thus far may partially be due to choice of stimulus materials or selection bias in participant groups. Finally, these studies are not conclusive about the mechanisms underlying misophonia, but do highlight candidate processes for further research in both central and peripheral nervous systems, including specific salience to particular stimuli, early effects in the auditory system, the importance of physical (bodily) sensations and responses, and the integration of perceptual salience with atypical awareness of internal body states.

## Self-report measures of misophonia and mental health

A small number of studies using self-report and interview measures have begun to characterize possible psychological and psychiatric correlates of misophonia. Many of these studies share as aims the need to characterize (a) the subjective experiences and responses to triggers in those with misophonia, (b) the relationship between misophonia and other conditions, and (c) whether misophonia should be considered unique and distinct from established psychiatric disorders. The first of these studies was by Schröder et al. (2013), who recruited 42 Dutch adults from a mental health clinic who self-reported misophonia symptoms. A psychiatrist interviewed participants to assess psychiatric diagnoses. Results suggest that participants met criteria for a wide range of co-occurring psychiatric disorders. Specifically, the majority met criteria for obsessive-compulsive personality disorder (OCPD; 52.4%), whereas others met criteria for mood disorders (7.1%), ADHD 4.8%, panic disorder (2.4%), and obsessive compulsive disorder (OCD 2.4%). In addition, participants also self-reported several characteristics about their responsivity to misophonic sounds, including: (1) aversive and angry feelings evoked by particular sounds, (2) rare potentially aggressive responses, (3) recognition by the misophonic individual that his/her behavior is excessive, (4) avoidance behavior, and (5) distress and interference in daily life.

Based on these results, Schröder et al. (2013) suggested the possibility that misophonia be considered a disorder under the broader classification Obsessive and Compulsive Related Disorders in the DSM-5 (American Psychiatric Association, 2013). However, the authors also stated that it is premature to make firm conclusions about this possibility. Indeed, when considering the sampling approach from one clinic, the small sample size, the use of a single diagnostic assessor, and the lack of inter-rater reliability reported for psychiatric diagnostic assessment, it is premature to conclude from this study whether misophonia is best categorized as an obsessive compulsive-related disorder. In addition, given that only 2.4% (*n* = 1) of the sample met criteria for OCD, and that most met criteria for OCPD, the results suggest that future research is needed to explore the co-occurrence of misophonia among those with OCPD symptoms.

Schröder et al. (2013) also suggest that misophonia be considered as a discrete psychiatric disorder. However, the results from their study do not clearly point to misophonia being a feature of any one psychiatric disorder. Indeed, limitations to the design of this study preclude conclusions about the classification of misophonia as a discrete psychiatric disorder. Additional research is needed using, for example, larger and more diverse samples with rigorous methods of assessment and data analysis before it is reasonable to conclude that misophonia is best classified as a discrete psychiatric disorder.

An additional consideration from Schröder et al. (2013) is the possible co-occurrence between misophonia and sensory over-responsivity, a syndromal subtype of sensory processing disorder (SPD). In noting the possible relationship between misophonia and SPD, the authors state that typical auditory sensitivity in SPD is only in response to loud and unexpected noises. However, research on sensory over-responsive children has not differentiated between loud or softer/patterned noises^3^. Therefore, associations between these two conditions warrants further exploration. More generally, results from Schröder et al. (2013) raise important questions about the expected co-occurrence between misophonia and other conditions. Indeed, the findings from this study help pave the way for future studies to investigate and further characterizing the relationship between misophonia and psychiatric disorders.

In another study, Wu et al. (2014) investigated the incidence, phenomenology, correlates, and level of impairment associated with misophonia symptoms in 483 undergraduate students through self-report measures. In this sample, nearly 20% of participants reported clinically significant misophonic symptoms, as measured by the Misophonia Questionnaire (MQ), a newly developed self-report inventory. Psychometric data suggest that the measure had high internal consistency, and preliminary convergent validity was reported by a significant correlation (*r* = 0.50) between misophonia symptoms and a self-report item assessing auditory over-responsivity from the Adult Sensory Questionnaire (Kinnealey and Oliver, 2002). Preliminary divergent validity was reported, as misophonia symptoms on the MQ were significantly positively correlated with sensory over-responsivity across other sensory domains (e.g., olfactory, *r* = 0.28; tactile, *r* = 0.34; visual, *r* = 0.33). In addition, higher symptoms on the MQ were positively correlated with measures of general life impairment and were moderately associated with obsessive-compulsive (*r* = 0.47), anxiety (*r* = 0.39), and depressive (*r* = 0.30) symptoms. The authors report that the positive correlations between misophonia symptoms and sensory sensitivities may indicate that misophonia symptoms are associated with a more general defensive motivational response across sensory systems.

In addition, the authors report that anxiety mediated the relationship between misophonia and anger outbursts^4^. This mediational finding, though preliminary and based on cross-sectional data, raises the possibility that the self-reported tendency to respond with anger may be a function of the magnitude of anxiety elicited by trigger cues. In other words, the experience and expression of anger may be a secondary emotional response following the elicitation of acute anxiety stemming from central defensive motivational system responses. To replicate and extend the mediational findings in Wu et al. (2014) future studies using prospective measurement should elucidate the dynamic unfolding over time of affective states and behavioral responses elicited by misophonic triggers. Limitations to this study notwithstanding (e.g., few male participants, reliance on self-report measures, use of undergraduates, preliminary validity and reliability data), results from Wu et al. (2014) provide initial psychometric data for the MQ as an appropriate self-report measure of misophonia symptoms.

In a recent replication and extension of Wu et al. (2014), Zhou et al. (2017) used the MQ to explore the relationship between misophonia and psychopathology in a sample of Chinese college students. Consistent with Wu et al. (2014), 17% of the sample (*N* = 415) reported misophonia symptoms caused clinically significant impairment in their daily lives. Higher MQ scores were significantly positively correlated with symptoms of anxiety, depression, and OCD. This suggests misophonia is not uniquely associated with anxiety, depression, or OCD *per se*, but may instead be more generally correlated with higher levels of psychological distress. Additionally, higher MQ scores were significantly positively correlated with general sensory defensiveness (i.e., over-responsivity) across visual, tactile, olfactory, and auditory sensory domains. This suggests that misophonia symptoms may be related to sensory over-responsivity in general, and not to auditory over-responsivity specifically.

In another recent study, Dozier and Robinson (2017) explored self-reported responses to misophonic cues presented via teleconferencing technology to a sample of 27 adults. Participants self-reported emotional responses and associated behavioral response tendencies to trigger sounds, including but not limited to clenching of the hands, jaw, shoulders, and chest. Most participants self-reported feeling anxiety (92.3%) or anger (92.3%), with approximately half indicating a desire to escape (53.8%) from the trigger sounds and a disgust (46.2%) response. Based on their findings, the authors suggest that misophonia symptoms are conditioned physical and emotional responses. In the absence of any experimental research on misophonia using conditioning paradigms, however, it is too early to make such a conclusion. Indeed, the role of conditioning and learning in the etiology and maintenance of misophonia needs to be elucidated.

Despite the limitations of a small sample size, lack of a control group, lack of psychiatric or clinical interviews, and the absence of rigorously controlled experimental conditions, this study helps informs the conceptualization of misophonia in several ways. For example, these findings provide empirical evidence that emotional responses to triggers cues are likely to include both subjective affective states and associated behavioral urges. Additionally, this study highlights the complexity of affective responses to misophonic cues, suggesting that the condition may not be only or best characterized by anger. Although more research is needed, one hypothesis is that activation of defensive motivational systems, which mediate behavior (e.g., fight-flight response) and affective states, more generally characterize misophonia than any one specific subjectively experienced affective state.

Rouw and Erfanian (2017) used an online recruitment approach to survey over 300 participants reporting misophonic complaints. Participants responded to questionnaire items assessing family and respondent history of misophonia, development of misophonia symptoms, and common responses to trigger sounds. Results revealed a pattern in the development of misophonia, with symptoms starting in childhood/early adulthood and increasing in intensity with repeated exposure to triggers. Approximately one third of the participants reported family members with misophonic symptoms. This finding underscores the need for research evaluating the environmental and genetic correlates of misophonia. In addition, there was diversity in the nature and intensity of misophonia symptoms, including heterogeneity in self-reported emotional, physical and cognitive responses to triggering stimuli. This suggests the possibility of individual differences in the underlying mechanisms of misophonic responses.

Half of the participants reported presence of autonomous sensory meridian responses characterized by “euphoric, relaxing, and tingling sensations with particular sounds or sights”. This is particularly interesting in the light of recent findings by Kumar et al. (2017) reporting atypical perception of internal body states in misophonia. Additionally, half of the participants in this study reported misophonic complaints without comorbidity with another condition. The other half reported having a variety of psychiatric conditions. Only one clinical condition showed a relationship to misophonia: the severity of misophonic complaints were stronger if the participants also reported having a diagnosis of post-traumatic stress disorder (PTSD).

Because there was no clear pattern of misophonia co-occurring with any psychiatric disorder across participants, the authors concluded that misophonia is a unique and independent condition. However, it is important to highlight that limitations in the study design (i.e., self-report via online data collection) precluded the collection of diagnostic information using structured interviews. In order for clearer conclusions to be made about the relationship between misophonia and psychiatric disorders, it is necessary to conduct research using psychometrically validated interview-based measures of psychiatric disorders.

McKay et al. (2017) also recently investigated the relationship between misophonia and psychopathology in a large sample using an online recruitment approach. In this study, participants with (*n* = 121) and without (*n* = 507) high levels of misophonia symptoms completed self-report instruments assessing clinical features related to OCD, GAD, body perception, anxiety sensitivity, distress tolerance, depression, dissociation, anger, behavioral inhibition/activation, and anxiety.

Using a multi-dimensional scaling approach, the authors reported obtaining a profile of clinical features that significantly accounted for 11% of the variance between those with and without high misophonia symptoms. This profile broadly was characterized by higher scores across most of the measures of psychopathology. More specifically, however, harm avoidance and ordering OCD symptoms were related to higher misophonia symptoms, whereas neutralizing, obsessions, and washing OCD symptoms were associated with lower misophonia symptoms. In addition, most the variance (70%) in the differences between groups with and without high symptoms of misophonia could not be attributed to any measures of psychopathology. This pattern of results suggests that misophonia may not be uniquely related to OCD, or to any specific psychiatric disorder.

Overall, results from studies using self-report measures collectively indicate that misophonia symptoms (a) can be measured using self-report instruments, (b) vary in phenotypic expression across individuals, and (b) do not appear to co-occur with any one specific psychiatric disorder. Misophonia has been observed across a wide variety of disorders (e.g., PTSD, OCPD), raising the question of whether it is a separate and unique condition. Similarly, findings across multiple studies using self-report methods converge to suggest that misophonia is correlated with higher psychological distress and psychopathology in general, but is not associated with any one specific disorder *per se*. The research to date is beginning to point to the possibility that there are clear and distinct characteristics which may set misophonia apart from psychiatric disorders. However, the exact nature of what characterizes misophonia symptoms differentially from existing psychiatric conditions remains unknown. Indeed, the studies described above using self-report suffer from limitations (e.g., self-report biases) shared by all studies using questionnaires and not using random sampling. Replication studies and larger studies using structured psychiatric diagnostic interviews will help to clarify whether and to what extent misophonia may be correlated with other psychiatric disorders, and whether there are symptoms differentiating misophonia best from other disorders.

## Cross-disciplinary perspectives

Beyond studies with a primary aim of investigating misophonia, other bodies of research can be drawn upon that may offer important insights. In particular, research examining auditory gating, sensory processing, and neural processes underlying these can add to a foundation for the cross-disciplinary conceptualization of misophonia. Although a comprehensive review of all potential bodies of research that may contribute to multidisciplinary conceptualization of misophonia is beyond the scope of this paper, we review these distinct areas, with some specific study examples.

## Auditory gating and sensory over responsivity

Previous research has been conducted with children and adults who have difficulty modulating sensory stimuli in a graded manner (Brown et al., 2001; Kisley et al., 2004; Davies and Gavin, 2007; Gavin et al., 2011). The sensory gating response is the brain's capacity to selectively regulate sensitivity to sensory stimuli (Yadon et al., 2009), and individuals gating impairments specific to the auditory modality have been studied across various samples (Jeste and Nelson, 2009). Examples include children and adults with autism spectrum disorder (Perry et al., 2007), SPD (Green and Ben-Sasson, 2010) and schizophrenia (McCarley et al., 1991; Brockhaus-Dumke et al., 2008). Previous research in this area consistently shows abnormal information processing, measured in terms of early ERP^5^ components in the sensory cortex (Brett-Green et al., 2010). These results are consistent with those from Schröder et al. (2014), and suggest that atypical sensory processing may be observed among adults with misophonia and children with sensory modulation impairments.

In addition, children characterized by heightened sensory over-responsivity have demonstrated an increased number, frequency and higher magnitude of SCR responses to sensory cues across sensory domains, as well as slower rates of habituation to such cues compared to typically developing control children (McIntosh et al., 1999). Sensory over-responsive children also demonstrate greater levels of baseline arousal and higher reactivity in response to various types of sensory stimuli than children with autism spectrum disorders, although children with autism have lower baseline arousal (Schoen et al., 2009).

Whereas SPD, like misophonia, is not delineated as a discrete DSM-5 disorder or ICD-10 condition, and has commonly received attention in the field of occupational therapy, auditory sensory over-responsivity shares some similar phenotypic signs and symptoms to those expressed by individuals with misophonia.

In addition, when one considers that many individuals with misophonia also report visual sensitivity to movement, it is appropriate to look at studies related to sensory over-responsivity across sensory domains in an effort to develop hypotheses about possible mechanisms underlying misophonia. Accordingly, it is reasonable and appropriate to consider the research on sensory over-responsivity as part of a broadly construed cross-disciplinary account of misophonia.

More specifically, although both Schröder and colleagues' work (Schröder et al., 2013) as well as studies of children with SPD (Davies and Gavin, 2007) demonstrate commonalities in auditory gating deficits, these comparisons should be made somewhat tentatively, as the sensory processing studies did not parse out responsivity to loud vs. soft/pattern based auditory stimuli, and misophonia studies have not deconstructed the complex misophonic trigger sounds, nor sampled this population in large enough studies to determine how much aversive sounds vary from one individual to another. In view of these studies that relate to auditory gating, it is logical when returning to the Jastreboffs' (Jastreboff and Jastreboff, 2001) original theory of misophonia as related to pattern-based sounds, to include repetition of auditory stimuli as a possible sound related variable associated with lack of habituation to trigger sounds and/or to consider individual differences with regard to auditory gating (or overall habituation) as a potential factor reactivity to specific sounds.

Studies examining noise sensitivity (NS) and their neural basis offer another cross-disciplinary avenue that may inform misophonia. In the context of NS, noise is any unwanted sound and the degree of aversive reaction to it defines NS. The aversive reaction in NS does not depend on the loudness of sound and in that sense shares a similarity with misophonia. A few studies in recent years have examined the neural basis of NS. Kliuchko et al. (2016) measured multi-feature mismatch negativity (MMN) along with evoked responses by the sound onsets. They found that magnitude of P1 response was smaller in subjects with higher NS reflecting that representation of sound in the central auditory system was compromised in NS individuals. This bears some resemblance to a study in misophonia by Schröder et al. (2014) who found that magnitude of N1 peak in misophonics was smaller than that in controls, which also is suggestive of sound encoding deficits in misophonia.

Kliuchko et al. (2016) suggest that the relatively recent concept in perception called predictive coding (PC; Pelt et al., 2016) could be offered as a theoretical explanation for their results. In PC, the brain generates hypotheses or a model based on expectations and predictions about what sensory stimuli are likely to be experienced (Seth, 2013). The hypotheses are continually processed and updated based on new information. Combining both top-down and bottom up neural processes, sensory stimuli are matched against the predictions; when the incoming information matches predictions, the prediction error is small, and when they do not match, error is high. It is possible that individuals with high NS (and thus, high uncertainty regarding sensory expectations) have impairments in the top-down encoding of sound features and, accordingly, difficulties predicting incoming sensory information. Thus, a low MMN response for participants with high NS is considered to be a high prediction error, and a neural marker of this phenomenon could be a suppressed P1 response (Friston, 2005; Stefanics et al., 2014). By turning to this literature, studies examining responses to misophonic trigger sounds may investigate hypotheses associated with the mechanisms underlying NS.

In a more recent study (Kliuchko et al., 2017), gray matter volume was measured in a number of sensory (e.g., Heschl gyrus, planum temporale) and emotion processing related areas (e.g., anterior insula, amygdala). Interestingly, a positive correlation between the volume and NS was measured in anterior insula, a region which has been shown to play a key role in misophonia (Kumar et al., 2017). Further collaborative work between the researchers studying misophonia and NS would be helpful to understand the similarities and differences between the two phenomena.

## Network level applications to misophonia

The conceptual framework of network level neural models may be useful in the development of a model specifying candidate neural mechanisms of misophonia. This approach emphasizes the understanding of basic brain processes at the network level (Bressler and Menon, 2010). Using a network level approach permits testable hypotheses about multiple spatially separated brain regions working in an integrated and coordinated manner. Although most of our understanding of how the brain implements perception, cognition and emotion processing is based on the assumption of assigning a unique role to each region of the brain, there is a growing realization that this approach is not fruitful in understanding brain function, as a given brain area can be involved in multiple functions. Instead, it has been proposed that functions of the brain should be understood at the network level (Bressler and Menon, 2010). At this level a number spatially separated brain regions coordinate and integrate to implement a function. In order to pinpoint the neural mechanisms behind misophonia, it will not be sufficient to determine which brain areas are abnormally active in misophonia, but also to understand how those brain areas work at a network level.

Specifically applied to misophonia, a network modeling approach may be used to elucidate underlying functional connectivity and neural pathways with impaired functioning across disparate regions of the brain. Kumar et al. (2012) demonstrated the neural representation of aversive, salient sounds perceived as unpleasant. Specifically, using functional magnetic resonance imaging fMRI. Kumar et al. (2012) revealed brain responses in the amygdala and the auditory cortex while listening to unpleasant sounds. Interestingly, this study revealed that the amygdala encodes information concerning the valence and acoustic features of sounds, and that these characteristics modulate the functional connectivity between the amygdala and auditory cortex. This has relevance to misophonia as it points to a potential mechanism via which the auditory cortex may become hyperactive in misophonia, possibly accounting for abnormal perceptual responses in those with misophonia. That is, it is possible that the parts of the brain which extract salience from (or assign negative or positive valence to) sounds may respond abnormally to the typical misophonia trigger sounds. A high level of salience and negative valence may then modulate the activity of sensory (auditory) cortex. In addition, neural circuits coordinating defensive motivation systems, including emotional processing (e.g., amygdala, insula, etc.) also likely are activated as part a broader cascade of neurobehavioral responses.

## Suggestions for a research agenda

In order to advance a scientific understanding of misophonia that also can be understood by the public and those who suffer from misophonia, it is important to use clear and consistent terminology. For example, although misophonia translates to “hatred of sound,” the phenotype of this syndrome does not appear to be limited to the experience and/or expression of anger alone. Indeed, the original conceptualization by Jastreboff and Jastreboff (2001) was one of decreased sound tolerance in which subconscious connections between auditory and emotional stimuli elicit misophonia symptoms maintained by principles and processes governing conditioning (e.g., associative learning and memory). From this perspective, conditioned responses may vary across individuals and contexts. One implication of this model is that anger is not a required affective response in misophonia. An important gap in the existing research is the need to precisely characterize the nature and dynamic temporal unfolding of affective, cognitive, and behavioral responses to misophonic triggers.

Research also could benefit from improved clarity and consistency in terminology in the description of neural processes associated with misophonia. For example, the term “limbic system” has been used to describe the central region responsible for mediating emotional responses in those with misophonia. However, this may be an overly simplistic explanation that is not well justified in light of contemporary affective neuroscientific research demonstrating the importance of models using varied and integrated processes across multiple areas of the brain. Put differently, and in the context of network level neural models, emotional processes take place in many areas and on many levels within the brain. LeDoux (2015), for example, describes an emotion as an elusive aggregate of many systems and functions in the nervous system that involve both conscious and unconscious (older and newer) brain structures. This is relevant both to the conceptualization of misophonia both diagnostically and in regard to how individuals with this form of decreased sound tolerance form attributions about their misophonic responses.

Since we do not have sufficient evidence to make conclusions about the role of genetics in misophonia, or to firmly conclude how this condition develops in regards to conditioning and associated neurobiological processes, we suggest avoiding language suggestive of a false dichotomy between nature and nurture. Describing disorders as “genetic” vs. “conditioned” gives way to a potentially false dichotomy that affects both diagnosis and treatment. Put differently, misophonia is a complex neurophysiological phenomenon. There are no scientific data to support claims that it is specifically the result of any single etiological factor or process. Because attention, learning, memory, emotion, cognition, and other basic processes germane to misophonia all are grounded in basic biological processes and influenced by environmental factors, the distinction between nature and nurture is not warranted.

There are several key areas of research needed to rapidly advance a scientific understanding of this complex phenomenon. Chief among these needs are studies that add critical data to characterize the public health significance of misophonia. For example, it is important to gain a more precise understanding of the clinical symptoms and features that occur among those who report impairment in functioning associated with misophonia. Case studies and preliminary research have begun to address this need, though such studies have used samples of limited size (Schröder et al., 2014) and generalizability (Wu et al., 2014). Empirical research is needed to clarify whether, for example, misophonia is a constellation of symptoms that can be classified categorically and discriminated from other related conditions and syndromes. Some researchers have suggested that because misophonia symptoms have not been found to correlate specifically with any single psychiatric disorder, perhaps misophonia should be conceptualized among a class of psychiatric disorders or as a discrete psychiatric disorder (Schröder et al., 2014). However, as has been noted by others (Taylor, 2017), we believe that there is inadequate scientific research to warrant clear conclusions about the exact nature of misophonia as a psychiatric disorder. Although the existing research does suggest that misophonia may not be better explained by an existing psychiatric disorder, whether misophonia is best classified as a discrete disorder or whether it is a syndrome that co-occurs with OCDs, personality disorders, anxiety disorders, or other psychiatric disorders will be determined through systematic research using multiple methods and measures. Until this occurs, it is suggested that conclusions from individual studies about how to classify misophonia be clearly identified as preliminary and used as hypotheses to test using rigorous research methods.

In addition, as part of investigating whether misophonia is best understood empirically as a categorical or dimensional phenotype, advancements are needed in the self-report measures used to collect information from patients. To date, there are several instruments that have been developed specifically for the study of misophonia (e.g., MQ) (Wu et al., 2014). As preliminary self-report inventories, these measures offer value as they have helped to begin characterizing the symptoms of misophonia. However, in order for the etiology, maintenance, and treatment of misophonia to be characterized rigorously using scientific methods, additional research using prospective methodologies is needed to further validate self-report inventories.

As self-report measures of misophonia symptoms continue to be developed and refined, epidemiologic studies exploring the prevalence and incidence of misophonia will need to be conducted. Such research will help to elucidate (a) the extent to which varying levels of misophonia symptom severity can be expected to occur in the general population, (b) normative differences in misophonia symptoms across age sex, gender, and other demographic factors, and (c) population level estimates of the developmental, medical, and psychiatric factors associated with misophonia. In addition, prospective epidemiologic studies would permit inferences about the relative contributions of genetic and environmental influences on the expression and development of misophonia over time.

In order to elucidate the neurophysiological mechanisms underlying misophonia, highly rigorous controlled research needs to be conducted using objective laboratory measures and appropriately powered sample sizes. As detailed above, preliminary research has investigated misophonia using affective and cognitive neuroscience methods. The work done by Kumar et al. (2012, 2017) and Schröder et al. (2014), for example, offers important insights into which neural systems are activated when misophonics hear certain trigger sounds. Outside the misophonia literature, extensive research has been conducted in animal and human studies to characterize the primary and secondary nervous system structures, functions, and functional connectivity governing defensive motivational responses to aversive auditory stimuli. We recommend that research with the primary aim of investigating the neural underpinnings of misophonia (a) be grounded in a theoretical framework offering testable hypotheses and (b) leverage previous research exploring systems subserving sensory processing, emotional reactivity, and the regulation of emotional arousal in clinical samples of individuals with heightened anxious arousal. It is essential that audiologists, basic scientists and behavioral health researchers work together in a cross-disciplinary manner using team-based science. This has the potential to more rapidly identify discoveries with near-term clinical significance than would occur if researchers use a more siloed model with narrow conceptualizations of misophonia as a phenomenon pertinent to one discipline or theoretical model.

Finally, the sounds that people with misophonia find aversive should be studied in great depth. This includes amplitude (loudness), pitch (or frequency of sound waves), and duration (time interval), and timber (a combination of frequency modulation, the rates of change in amplitude, and elements of harmony). A better understanding of the specific sound features, including studies of reactivity when sounds are presented in a bimodal and unimodal manner (sound without visuals vs. sound with visuals) as in the Edelstein et al. (2013) study, would be an important next step in research. Studying sound features and relating them to different kinds of auditory processing, as well as visual/auditory integrative processing, will better inform the research related to reactivity, and help to define both the population and the disorder. Following this line of research in a logical order, rather than first comparing misophonia to other disorders at a time in which science still redefines brain functioning and the nature of psychiatric and neurological disorders, would likely be more prudent.

## Clinical considerations

To date, no randomized controlled trials evaluating treatments for misophonia have been published. Case studies (e.g., Bernstein et al., 2013; Webber et al., 2014; McGuire et al., 2015; Schneider and Arch, 2017) and an uncontrolled clinical trial (Schröder et al., 2017) using cognitive and behavioral interventions have been reported. There are no published studies reporting the effects of pharmacological treatments. In the absence of any empirically supported treatments for misophonia, how can healthcare providers provide care to individuals who are suffering with these symptoms? We believe there are several reasonable approaches that clinicians can take when approached by patients or providers about interventions for misophonia. First, providers need to ethically characterize the state of empirical research on treatments to patients. As an example, it is recommended that providers inform patients that there are few assessment measures to quantify misophonia symptoms, a lack of clarity in the etiology of misophonia, and no known treatments shown to work in controlled randomized trials. Second, providers can educate patients about the uncertainty with regard to how to categorize misophonia. It is inappropriate at this point, in the absence of sufficient research, to define misophonia as a psychiatric disorder. Similarly, despite the preliminary research identifying psychiatric diagnostic correlates, it remains speculative to describe misophonia as a feature or co-occurring syndrome related to any specific psychiatric disorder or class of disorders.

Without empirical support to characterize the treatment of misophonia, a third clinical consideration is that providers adopt a multi-disciplinary approach to the assessment and intervention of misophonia and co-occurring physical and behavioral health problems (Meltzer and Herzfeld, 2014). Such an approach would benefit from being individualized, such that each patient's unique history and symptoms be carefully considered by professionals across appropriate fields, such as neurology, audiology, occupational therapy, neuropsychology, psychiatry, and clinical psychology. Further, it is recommended that such a multi-disciplinary approach utilize a team-based approach with a shared electronic medical record and regular team meetings. This general framework can be described as a multi-disciplinary misophonia care management model.

Using a team-based management model, care pathways for misophonia can be delineated and individualized such that, for example, patients with co-occurring neurological conditions may receive appropriate care concurrent with care these same individuals might receive from a psychologist using behavioral methods to improve response patterns to misophonic triggers. Alternately, as a different example, a higher functioning patient with misophonia and co-occurring generalized sensory over-responsivity could receive sensory integration interventions with supportive counseling and education from an occupational therapist. For that patient, a psychiatric or psychological intervention may not be needed. However, for some patients with more severe misophonia, the care pathway might include neuropsychological testing, occupational therapy, psychotropic medication, and/or empirically supported behavioral interventions shown to help reduce anxiety, anger, or other outcomes.

For patients with functional impairments and psychological distress associated with misophonia, it is recommended that the multi-disciplinary care pathway provide coping skills for patients to learn to prevent and respond more effectively to their symptoms. Contemporary cognitive behavioral therapies offer empirically supported principles of change that, although not tested directly for misophonia in controlled clinical trials, may be considered as reasonable strategies to use in a coping skills-based approach. Examples include acceptance- [e.g., mindfulness, cognitive defuse (Hayes et al., 1999; Kabat-Zinn, 2009); distress tolerance skills (Linehan, 2015) and change-based behavioral (e.g., interpersonal skills) and cognitive (e.g., cognitive reappraisal) interventions]. However, these skills utilized in isolation may not be enough. Family counseling, practical strategies for knowing when and when not to avoid stimuli, as well as individually-driven methods for down-regulating the nervous system should also be included.

Clinical guidelines for the management of misophonia should emphasize the use of coping skills grounded in empirically supported principles of change, as there is no evidence-based behavioral or pharmacological treatment shown to be efficacious for misophonia. While the study of misophonia is beginning, a clinician would be remiss to claim that any particular treatment is “standard” or “tested” or validated. However, this population includes sufferers, including children and adults, and we recommend that coping skills used to manage misophonia be derived across disciplines such as psychology, psychiatry, audiology, occupational therapy and neurology. Clinicians from each discipline can work together in cross-disciplinary teams to implement individualized coping skills plans for clients/patients. Using this approach, we recommend that clinicians from unique disciplines and training backgrounds work together to educate each other about the neurophysiological, emotional, cognitive, and behavior manifestations of misophonia. Through a collaborative and team-based model, clinical interventions and treatment plans can be thoughtfully tailored to each individual, with empirically supported principles of change (i.e., cognitive, emotional, behavioral, physiological) used until treatments shown to work through rigorous scientific testing are developed.

## Author contributions

All author contributed to the intellectual development and writing of this manuscript. JB and MR conceptualized, wrote, revised, and synthesized revisions of this manuscript. LM, RR, SK, MEr, MEd, MM wrote sections and helped with revisions of the manuscript.

### Conflict of interest statement

The authors declare that the research was conducted in the absence of any commercial or financial relationships that could be construed as a potential conflict of interest.

## Footnotes

^1^Unisensory (visual or auditory alone).

^2^Multisensory (visual and auditory together).

^3^On many of the SPD/SOR scales there are items that include both loud noises and repetitive noises (see SPDfoundation.net).

^4^Notably, studies that include the relationship of SOR and anxiety (Ben-Sasson et al., 2009b, 2010; Lane et al., 2010, 2012) may inform how anxiety mediates misophonia in general and in regard to anger outbursts.

^5^ERP (event related potential) refers to positive and negative voltage fluctuations of the brain in response to stimuli as measured by an electroencephalogram (EEG).

## References

[B1] American Psychiatric Association (2013). Diagnostic and Statistical Manual of Mental Disorders, 5th Edn. Arlington, VA: American Psychiatric Publishing.

[B2] Attias J. Bleich A. Furman V. Zinger Y. (1996). Event-related potentials in post-traumatic stress disorder of combat origin. Biol. Psychiatry 40, 373–381. 10.1016/0006-3223(95)00419-X 8874838

[B3] Baguley D. M. McFerran D. J. (2011). Hyperacusis and disorders of loudness perception, in Textbook of Tinnitus, eds Møller A. R. Langguth B. DeRidder D. Kleinjung T. (New York, NY: Springer), 13–23.

[B4] Baguley J. (2016). Outsourcing Clinical Development: Strategies for Working with CROs and Other Partners. New York, NY: CRC Press.

[B5] Ben-Sasson A. Carter A. S. Briggs-Gowan M. J. (2009b). Sensory over-responsivity in elementary school: prevalence and social-emotional correlates. J. Abnorm. Child Psychol. 37, 705–716. 10.1007/s10802-008-9295-8 19153827 PMC5972374

[B6] Ben-Sasson A. Carter A. S. Briggs-Gowan M. J. (2010). The development of sensory over-responsivity from infancy to elementary school. J. Abnorm. Child Psychol. 38, 1193–1202. 10.1007/s10802-010-9435-9 20623174

[B7] Ben-Sasson A. Hen L. Fluss R. Cermak S. A. Engel-Yeger B. Gal E. (2009a). A meta-analysis of sensory modulation symptoms in individuals with autism spectrum disorders. J. Autism Dev. Disord. 39, 1–11. 10.1007/s10803-008-0593-3 18512135

[B8] Bernstein R. E. Angell K. L. Dehle C. M. (2013). A brief course of cognitive behavioural therapy for the treatment of misophonia: a case example. Cogn. Behav. Ther. 6:e10 10.1017/S1754470X13000172

[B9] Brang D. Hubbard E. M. Coulson S. Huang M. Ramachandran V. S. (2010). Magnetoencephalography reveals early activation of V4 in grapheme-color synesthesia. Neuroimage 53, 268–274. 10.1016/j.neuroimage.2010.06.008 20547226

[B10] Brang D. Rouw R. Ramachandran V. S. Coulson S. (2011). Similarly shaped letters evoke similar colors in grapheme–color synesthesia. Neuropsychologia 49, 1355–1358. 10.1016/j.neuropsychologia.2011.01.002 21219918

[B11] Bressler S. L. Menon V. (2010). Large-scale brain networks in cognition: emerging methods and principles. Trends Cogn. Sci. 14, 277–290. 10.1016/j.tics.2010.04.004 20493761

[B12] Brett-Green B. A. Miller L. J. Schoen S. A. Nielsen D. M. (2010). An exploratory event-related potential study of multisensory integration in sensory over-responsive children. Brain Res. 1321, 67–77. 10.1016/j.brainres.2010.01.043 20097181

[B13] Brockhaus-Dumke A. Mueller R. Faigle U. Klosterkoetter J. (2008). Sensory gating revisited: relation between brain oscillations and auditory evoked potentials in schizophrenia. Schizophrenia Res. 99, 238–249. 10.1016/j.schres.2007.10.034 18160261

[B14] Brown C. Tollefson N. Dunn W. Cromwell R. Filion D. (2001). The adult sensory profile: measuring patterns of sensory processing. Am. J. Occup. Ther. 55, 75–82. 10.5014/ajot.55.1.75 11216370

[B15] Cabranes J. A. Ancín I. Santos J. L. Sánchez-Morla E. García-Jiménez M. A. Rodríguez-Moya L.. (2013). P50 sensory gating is a trait marker of the bipolar spectrum. Eur. Neuropsychopharmacol. 23, 721–727. 10.1016/j.euroneuro.2012.06.008 22770636

[B16] Cavanna A. E. Seri S. (2015). Misophonia: current perspectives. Neuropsychiatr. Dis. Treat. 11, 2117–2123. 10.2147/NDT.S81438 26316758 PMC4547634

[B17] Colizoli O. Murre J. M. Rouw R. (2013). A taste for words and sounds: a case of lexical-gustatory and sound-gustatory synesthesia. Front Psychol. 4:775. 10.3389/fpsyg.2013.00775 24167497 PMC3806228

[B18] Danesh A. A. Kaf W. A. (2012). DPOAEs and contralateral acoustic stimulation and their link to sound hypersensitivity in children with autism. Int. J. Audiol. 51, 345–352. 10.3109/14992027.2011.626202 22299666

[B19] Davies P. L. Gavin W. J. (2007). Validating the diagnosis of sensory processing disorders using EEG technology. Am. J. Occup. Ther. 61, 176–189. 10.5014/ajot.61.2.176 17436840

[B20] Dozier T. H. (2015). Etiology, composition, development and maintenance of misophonia: a conditioned aversive reflex disorder. Psychol. Thought 30, 114–129. 10.5964/psyct.v8i1.132

[B21] Dozier T. H. Robinson K. L. (2017). Phenomenology of misophonia: initial physical and emotional responses. Am. J. Psychol. 130, 431–438. 10.5406/amerjpsyc.130.4.0431

[B22] Edelstein M. Brang D. Rouw R. Ramachandran V. S. (2013). Misophonia: physiological investigations and case descriptions. Front. Hum. Neurosci. 7:296. 10.3389/fnhum.2013.00296 23805089 PMC3691507

[B23] Ferreira G. M. Harrison B. J. Fontenelle L. F. (2013). Hatred of sounds: misophonic disorder or just an underreported psychiatric symptom? Ann. Clin. Psychiatry 25, 271–274. 24199217

[B24] Finsterwald C. Alberini C. M. (2014). Stress and glucocorticoid receptor-dependent mechanisms in long-term memory: from adaptive responses to psychopathologies. Neurobiol. Learn. Mem. 112, 17–29. 10.1016/j.nlm.2013.09.017 24113652 PMC3979509

[B25] Friston K. (2005). A theory of cortical responses. Philos. Trans. R. Soc. Lond. B Biol. Sci. 360, 815–836. 10.1098/rstb.2005.1622 15937014 PMC1569488

[B26] Gavin W. J. Dotseth A. Roush K. K. Smith C. A. Spain H. D. Davies P. L. (2011). Electroencephalography in children with and without sensory processing disorders during auditory perception. Am. J. Occup. Ther. 65, 370–377. 10.5014/ajot.2011.002055 21834451

[B27] Green S. A. Ben-Sasson A. (2010). Anxiety disorders and sensory over-responsivity in children with autism spectrum disorders: is there a causal relationship? J. Autism Dev. Disord. 40, 1495–1504. 10.1007/s10803-010-1007-x 20383658 PMC2980623

[B28] Hayes S. C. Strosahl K. D. Wilson K. G. (1999). Acceptance and Commitment Therapy: An Experiential Approach to Behavior Change. New York, NY: Guilford Press.

[B29] Hazell J. W. Jastreboff P. J. (1990). Tinnitus. I: auditory mechanisms: a model for tinnitus and hearing impairment. J. Otolaryngol. 19, 1–5. 2179573

[B30] Jastreboff M. M. Jastreboff P. J. (2001). Components of decreased sound tolerance: hyperacusis, misophonia, phonophobia. ITHS News Lett. 2, 5–7.

[B31] Jastreboff M. M. Jastreboff P. J. (2002). Decreased sound tolerance and tinnitus retraining therapy (TRT). Aust. NZ J. Audiol. 24, 74–84. 10.1375/audi.24.2.74.31105

[B32] Jastreboff P. J. (1990). Phantom auditory perception (tinnitus): mechanisms of generation and perception. Neurosci. Res. 8, 221–254. 10.1016/0168-0102(90)90031-9 2175858

[B33] Jastreboff P. J. Gray W. C. Gold S. L. (1996). Neurophysiological approach to tinnitus patients. Otol. Neurotol. 17, 236–240. 8723954

[B34] Jastreboff P. J. Jastreboff M. M. (2014). Treatments for decreased sound tolerance (hyperacusis and misophonia), in Seminars in Hearing, Vol. 35 (New York, NY: Thieme Medical Publishers), 105–120.

[B35] Jeste S. S. Nelson C. A. III. (2009). Event related potentials in the understanding of autism spectrum disorders: an analytical review. J. Autism Dev. Disord. 39, 495–510. 10.1007/s10803-008-0652-9 18850262 PMC4422389

[B36] Johnson P. L. Webber T. A. Wu M. S. Lewin A. B. Murphy T. K. Storch E. A. (2013). When selective audiovisual stimuli become unbearable: a case series on pediatric misophonia. Neuropsychiatry 3, 569–575. 10.2217/npy.13.70

[B37] Kabat-Zinn J. (2009). Full Catastrophe Living: Using the Wisdom of Your Body and Mind to Face Stress, Pain, and Illness. New York, NY: Delta.

[B38] Kinnealey M. Oliver B. (2002). Adult Sensory Questionnaire. Unpublished raw data. Temple University, College of Allied Health Professionals, Department of Occupational Therapy 3307.

[B39] Kisley M. A. Noecker T. L. Guinther P. M. (2004). Comparison of sensory gating to mismatch negativity and self-reported perceptual phenomena in healthy adults. Psychophysiology 41, 604–612. 10.1111/j.1469-8986.2004.00191.x 15189483

[B40] Kliuchko M. Heinonen-Guzejev M. Vuust P. Tervaniemi M. Brattico E. (2016). A window into the brain mechanisms associated with noise sensitivity. Sci. Rep. 6:39236. 10.1038/srep39236 27976708 PMC5157031

[B41] Kliuchko M. Puoliväli T. Heinonen-Guzejev M. Tervaniemi M. Toiviainen P. Sams M.. (2017). Neuroanatomical substrate of noise sensitivity. Neuroimage 167, 309–315. 10.1016/j.neuroimage.2017.11.041 29175201

[B42] Kluckow H. Telfer J. Abraham S. (2014). Should we screen for misophonia in patients with eating disorders? A report of three cases. Int. J. Eat. Disord. 47, 558–561. 10.1002/eat.22245 24431300

[B43] Kumar S. Hancock O. T. Sedley W. Winston J. S. Callaghan M. F. Allen M.. (2017). The brain basis for misophonia. Curr. Biol. 27, 527–533. 10.1016/j.cub.2016.12.048 28162895 PMC5321671

[B44] Kumar S. von Kriegstein K. Friston K. Griffiths T. D. (2012). Features versus feelings: dissociable representations of the acoustic features and valence of aversive sounds. J. Neurosci. 32, 14184–14192. 10.1523/JNEUROSCI.1759-12.2012 23055488 PMC3505833

[B45] Lane S. J. Reynolds S. Dumenci L. (2012). Sensory overresponsivity and anxiety in typically developing children and children with autism and attention deficit hyperactivity disorder: cause or coexistence? Am. J. Occup. Ther. 66, 595–603. 10.5014/ajot.2012.004523 22917126

[B46] Lane S. J. Reynolds S. Thacker L. (2010). Sensory over-responsivity and ADHD: differentiating using electrodermal responses, cortisol, and anxiety. Front. Integr. Neurosci. 4:8. 10.3389/fnint.2010.00008 20556242 PMC2885866

[B47] LeDoux J. (2015). Anxious: Using the Brain to Understand and Treat Fear and Anxiety. New York, NY: Penguin.

[B48] Linehan M. M. (2015). DBT Skills Training Manual. New York, NY: Guilford Publications.

[B49] McCarley R. W. Faux S. F. Shenton M. E. Nestor P. G. Adams J. (1991). Event-related potentials in schizophrenia: their biological and clinical correlates and new model of schizophrenic pathophysiology. Schizophrenia Res. 4, 209–231. 10.1016/0920-9964(91)90034-O 2039762

[B50] McGuire J. F. Wu M. S. Storch E. A. (2015). Cognitive-behavioral therapy for 2 youths with misophonia. J. Clin. Psychiatry 76, 573–574. 10.4088/JCP.14cr09343 26035184

[B51] McIntosh D. N. Miller L. J. Shyu V. Hagerman R. J. (1999). Sensory-modulation disruption, electrodermal responses, and functional behaviors. Dev. Med. Child Neurol. 41, 608–615. 10.1017/S0012162299001267 10503919

[B52] McKay D. Kim S. K. Mancusi L. Storch E. A. Spankovich C. (2017). Profile analysis of psychological symptoms associated with misophonia: a community sample. Behav. Ther. [Epub ahead of print]. 10.1016/j.beth.2017.07.002 29530266

[B53] Meltzer J. Herzfeld M. (2014). Tinnitus, hyperacusis, and misophonia toolbox, in Seminars in Hearing, Vol. 35 (New York, NY: Thieme Medical Publishers), 121–130.

[B54] Møller A. R. (2011). Misophonia, phonophobia, and ‘exploding head’ syndrome, in Textbook of Tinnitus, eds Møller A. R. Langguth B. DeRidder D. Kleinjung T. (New York, NY: Springer), 25–27.

[B55] Neal M. Cavanna A. E. (2012). P3 selective sound sensitivity syndrome (misophonia) and Tourette syndrome. J. Neurol. Neurosurg. Psychiatry 83, e1–e1. 10.1136/jnnp-2012-303538.20 23487200

[B56] Neal M. Cavanna A. E. (2013). Selective sound sensitivity syndrome (misophonia) in a patient with Tourette syndrome. J. Neuropsychiatr. Clin. Neurosci. 25, E01–E01. 10.1176/appi.neuropsych.11100235 23487200

[B57] Pelt S. V. Heil L. Kwisthout J. H. Ondobaka S. van Rooij I. J. Bekkering H. (2016). Beta-and gamma-band activity reflect predictive coding in the processing of causal events. Soc. Cogn. Affect. Neurosci. 11, 973–980. 10.1093/scan/nsw017 26873806 PMC4884316

[B58] Perry W. Minassian A. Lopez B. Maron L. Lincoln A. (2007). Sensorimotor gating deficits in adults with autism. Biol. Psychiatry 61, 482–486. 10.1016/j.biopsych.2005.09.025 16460695

[B59] Pienkowski M. Tyler R. S. Roncancio E. R. Jun H. J. Brozoski T. Dauman N.. (2014). A review of hyperacusis and future directions: part II. Measurement, mechanisms, and treatment. Am. J. Audiol. 23, 420–36. 10.1044/2014_AJA-13-0037 25478787

[B60] Raichle M. E. MacLeod A. M. Snyder A. Z. Powers W. J. Gusnard D. A. Shulman G. L. (2001). A default mode of brain function. Proc. Natl. Acad. Sci. U.S.A. 98, 676–682. 10.1073/pnas.98.2.676 11209064 PMC14647

[B61] Ramachandran V. S. Brang D. (2008). Tactile-emotion synesthesia. Neurocase 14, 390–399. 10.1080/13554790802363746 18821168

[B62] Rosenthal M. Z. Neacsiu A. D. Geiger P. J. (2016). Emotional reactivity to personally-relevant and standardized sounds in borderline personality disorder. Cogn. Ther. Res. 40, 314–327. 10.1007/s10608-015-9736-y

[B63] Rouw R. Erfanian M. (2017). A large-scale study of misophonia. J. Clin. Psychol. [Epub ahead of print]. 10.1002/jclp.22500 28561277

[B64] Rouw R. Scholte H. S. (2007). Increased structural connectivity in grapheme-color synesthesia. Nat. Neurosci. 10, 792–797 10.1038/nn1906 17515901

[B65] Saenz M. Koch C. (2008). The sound of change: visually-induced auditory synesthesia. Curr. Biol. 18, R650–R651. 10.1016/j.cub.2008.06.014 18682202

[B66] Schneider R. L. Arch J. J. (2017). Case study: a novel application of mindfulness-and acceptance-based components to treat misophonia. J. Context. Behav. Sci. 6, 221–225. 10.1016/j.jcbs.2017.04.003

[B67] Schoen S. A. Miller L. J. Brett-Green B. A. Nielsen D. M. (2009). Physiological and behavioral differences in sensory processing: a comparison of children with autism spectrum disorder and sensory modulation disorder. Front. Integr. Neurosci. 3:29. 10.3389/neuro.07.029.2009 19915733 PMC2776488

[B68] Schröder A. E. Vulink N. C. van Loon A. J. Denys D. A. (2017). Cognitive behavioral therapy is effective in misophonia: an open trial. J. Affect. Disord. 217, 289–294. 10.1016/j.jad.2017.04.017 28441620

[B69] Schröder A. van Diepen R. Mazaheri A. Petropoulos-Petalas D. de Amesti V. Vulink N.. (2014). Diminished n1 auditory evoked potentials to oddball stimuli in misophonia patients. Front. Behav. Neurosci. 8:123. 10.3389/fnbeh.2014.00123 24782731 PMC3988356

[B70] Schröder A. Vulink N. Denys D. (2013). Misophonia: diagnostic criteria for a new psychiatric disorder. PLoS ONE 8:e54706. 10.1371/journal.pone.0054706 23372758 PMC3553052

[B71] Seeley W. W. Menon V. Schatzberg A. F. Keller J. Glover G. H. Kenna H.. (2007). Dissociable intrinsic connectivity networks for salience processing and executive control. J. Neurosci. 27, 2349–2356. 10.1523/JNEUROSCI.5587-06.2007 17329432 PMC2680293

[B72] Seth A. K. (2013). Interoceptive inference, emotion, and the embodied self. Trends Cogn. Sci. 17, 565–573. 10.1016/j.tics.2013.09.007 24126130

[B73] Stansfeld S. A. Clark C. R. Jenkins L. M. Tarnopolsky A. (1985). Sensitivity to noise in a community sample: I. Measurement of psychiatric disorder and personality. Psychol. Med. 15, 243–254. 10.1017/S0033291700023527 4023129

[B74] Stefanics G. Kremlácek J. Czigler I. (2014). Visual mismatch negativity: a predictive coding view. Front. Hum. Neurosci. 8:666. 10.3389/fnhum.2014.00666 25278859 PMC4165279

[B75] Sullivan J. C. Miller L. J. Nielsen D. M. Schoen S. A. (2013). The presence of migraines and its association with sensory hyperreactivity and anxiety symptomatology in children with autism spectrum disorder. Autism 18, 743–747. 10.1177/1362361313489377 24072661

[B76] Taylor S. (2017). Misophonia: a new mental disorder?. Med. Hypotheses 103, 109–117. 10.1016/j.mehy.2017.05.003 28571795

[B77] Tyler R. S. Pienkowski M. Roncancio E. R. Jun H. J. Brozoski T. Dauman N.. (2014). A review of hyperacusis and future directions: part I. Definitions and manifestations. Am. J. Audiol. 23, 402–419. 10.1044/2014_AJA-14-0010 25104073

[B78] Ward J. Simner J. (2003). Lexical-gustatory synaesthesia: linguistic and conceptual factors. Cognition 89, 237–261. 10.1016/S0010-0277(03)00122-7 12963263

[B79] Webber T. A. Johnson P. L. Storch E. A. (2014). Pediatric misophonia with comorbid obsessive–compulsive spectrum disorders. Gen. Hosp. Psychiatry 36, 231-e1. 10.1016/j.genhosppsych.2013.10.018 24333158

[B80] Wu M. S. Lewin A. B. Murphy T. K. Storch E. A. (2014). Misophonia: incidence, phenomenology, and clinical correlates in an undergraduate student sample. J. Clin. Psychol. 70, 994–1007. 10.1002/jclp.22098 24752915

[B81] Yadon C. A. Bugg J. M. Kisley M. A. Davalos D. B. (2009). P50 sensory gating is related to performance on select tasks of cognitive inhibition. Cogn. Affect. Behav. Neurosci. 9, 448–458. 10.3758/CABN.9.4.448 19897797 PMC2810148

[B82] Zhou X. Wu M. S. Storch E. A. (2017). Misophonia symptoms among Chinese university students: incidence, associated impairment, and clinical correlates. J. Obsess. Compuls. Relat. Disord. 14, 7–12. 10.1016/j.jocrd.2017.05.001

